# Tonsillar Cough

**Published:** 1897-06

**Authors:** 


### TONSILLAR COUGH.

Furet in Archiv. Internat. de Laryngol.

Cough in general is a reflex provoked by some sort of an irritation at the site of the larynx. The initial focus of the irritation may be at quite a distant point and may arise in the tonsils.

The complex innervation of the latter amply explains this. The glosso-pharyngeal, lingual, spinal, and pneumo-gastric filaments all enter into a plexus on the surface of the tonsil. The first-named nerve has the most abundant distribution, especially on the surface, while the twigs of the others are rather distributed to the interior. Also the tonsil is encircled by muscles which are in very close relation with the muscular apparatus of the larynx.

Tonsillar cough may be evoked by acute and chronic inflammations of the gland by calculi, foreign bodies, mycosis, etc., and even by mere contact with the stylet. Adherences to the faucial pillars may also produce it. Sometimes the act of swallowing will remove the impulse to cough.

The tonsillar cough is violent, irregular, spasmodic, even suffocating. Various other reflexes, including lachrymation, may accompany it. There is no expectoration.

Cocaine will stop it temporarily if applied vigorously to the tonsillar surface.

Treatment is, of course, the restoration of the tonsillar site to a normal condition, and removal of the gland if of any size. The usual cough remedies are naturally without effect.

